# Supplementary material for: Investigating the optimal reactive balance training intensity in people with chronic stroke: Study protocol for a randomized control trial
Source: PLoS One. 2025 Sep 9;20(9):e0327937. doi: 10.1371/journal.pone.0327937 (PMC12419664; doi:10.1371/journal.pone.0327937)
Supplement: S2 File — (PDF) [file pone.0327937.s002.PDF]

Optimal intensity of reactive balance training post-stroke: a randomized controlled trial

Principal investigator
Avril Mansfield, R. Kin, PhD
Senior Scientist, KITE-Toronto Rehabilitation Institute, UHN
550 University Ave, Toronto, ON, M5G 2A2
avril.mansfield@uhn.ca
416-597-3422 ext 7831

Study personnel
Cynthia Danells, MSc, BScPT
Clinical Research Coordinator, KITE-Toronto Rehabilitation Institute, UHN
cynthia.danells@uhn.ca
416-597-3422 ext 3111

David Jagroop, MHSc., CSEP-CEP
Clinical Research Analyst, KITE-Toronto Rehabilitation Institute
david.jagroop@uhn.ca
(416) 597-3422 ext 7614

Tawanda Nigel Majoni, MSc
Graduate student, Rehabilitation Sciences Institute

Co-investigators
Elizabeth Inness, BScPT, MSc, PhD
Affiliate Scientist, KITE-Toronto Rehabilitation Institute, UHN
liz.inness@uhn.ca

LIST OF ABBREVIATIONS

ANCOVA: Analysis of covariance
CTCAE: Common terminology criteria for adverse events
EQ-5D-5L: EuroQoL health status measure, 5 dimensions, 5 levels
FES-I: Falls Efficacy Scale – International
ICC: Intraclass correlation coefficient
Mini-BEST: Mini Balance Evaluation Systems Test
RBT: Reactive balance training

1. INTRODUCTION

1.1 Background and rationale

Remaining active after stroke is essential to recovery, maintaining quality of life, and reducing secondary stroke risk.[1] However, impaired balance control post-stroke increases fall risk, contributes to fear of falling,[2] and reduces overall mobility, activity, and community integration.[3,4] Therefore, effective interventions to improve balance control and reduce fall risk are essential to ongoing recovery and quality of life post-stroke.[5] However, while there is unequivocal evidence that exercise, specifically balance training, is the most effective intervention for preventing falls in older adults without stroke,[6,7] it is unclear if exercise prevents falls after stroke.[8]

Effective balance reactions (e.g., reactive stepping) are essential to avoid falling following a loss of balance, or balance perturbation.[9] Reactive balance training (RBT), where clients experience repeated balance perturbations,[10,11] is a novel type of exercise that aims to improve control of balance reactions. We found that RBT improves reactive stepping ability in people with stroke.[12-15] Our recent meta-analysis found that RBT reduces rate of falls in daily life by ~40% among older adults and people with neurologic conditions, including stroke.[16]

Clinicians are unsure how to optimally prescribe RBT.[17,18] Exercise is often prescribed considering frequency, intensity, time, and type;[19] these parameters are interdependent. For example, as intensity increases, frequency or time can decrease to achieve similar benefits.[20,21] Clinicians report that limited time in rehabilitation services and competing rehabilitation goals and priorities mean that there is little time available to include RBT in a client's treatment plan.[17,18] Therefore, it would be valuable to know if a short duration of high-intensity RBT can improve reactive balance control post-stroke. However, it is possible that people with stroke would not tolerate high intensity perturbations and, therefore, require lower-intensity but long-duration RBT. No study has directly compared different RBT training intensities among people with stroke.

1.2 Objectives and hypotheses

The purpose of this study is to determine the optimal intensity of RBT post-stroke. The 'optimal' intensity is the intensity that improves reactive balance control in fewer sessions, without any apparent negative consequences (i.e., no increase in adverse outcomes). We expect that high-intensity training (50% above the multi-step threshold) will improve reactive stepping ability faster than moderate-intensity training (at the multi-step threshold). If the first reactive step is not effective in avoiding a fall after a loss of balance, additional steps must be taken;[22] therefore, the number of steps needed to recover from a loss of balance is a global indictor of the effectiveness of balance reactions.[23,24] We will assess reactions to novel, untrained, balance perturbations throughout training, and calculate the rate of decline in average number of steps taken to recover balance (i.e., learning rate). Retention of learning will be assessed one week post-training. Our primary hypotheses are:

1) Adaptation rate will be faster for high-intensity RBT than moderate-intensity RBT;
2) Adaptation rate will be faster for both RBT groups than a walking control group; and
3) Both RBT groups will have better retention of learning than the walking control group; i.e., the RBT groups will take fewer steps to respond to the novel perturbation than the walking control group one week post-training.

Secondary objectives are to compare the rate of adverse outcomes between groups, and to determine the effect of different intensities of RBT on the mechanisms underlying improved reactive stepping ability, functional balance, falls efficacy, and participation in daily activities.

2. METHODS TO BE USED

2.1 Trial design

This study is an outcome-adjudicator blinded randomized controlled trial, following motor learning protocols (Figure 1).[25,26] People with chronic stroke (n=63) will be randomly assigned to: 1) high-

intensity RBT, 2) moderate-intensity RBT, or 3) a walking control group. Training sessions will take place on four consecutive days; reactive stepping will be assessed pre-training, at the end of each training session (to determine the rate of adaptation), and after a delay of one week (to determine retention of learning effects) using a novel untrained perturbation. Falls in daily life, physical activity, and participation will be assessed for 12 months post-training.

Baseline<br>*Day 1*	Training sessions<br>*Days 2-5*	Retention<br>*Day 12*	Falls monitoring<br>*0-12 months post-training*	Follow-up<br>*12 months post-training*
Multi-step threshold<br>3 backward-fall perturbations<br>Mini-BEST, FES-I, EQ-5D-5L, fear of falling<br>Cohort descriptors	High-intensity RBT<br>3 backward-fall perturbations<br><br>Moderate-intensity RBT<br>3 backward-fall perturbations<br><br>Walking practice<br>3 backward-fall perturbations	3 backward-fall perturbations<br>Mini-BEST, FES-I, EQ-5D-5L, fear of falling	Falls in daily life	FES-I, EQ-5D-5L, fear of falling

Figure 1: Overview of the trial design. Mini-BEST=mini-Balance Evaluation Systems Test; FES-I=Falls Efficacy Scale-International; EQ-5D-5L=EuroQoL 5-level quality of life measure; RBT=reactive balance training

2.2 Trial interventions

Training sessions will be overseen by a physiotherapist and will last for 1 hour over 4 consecutive days. Training will occur using the 'FallsLab' custom-built 6m x 3m motion platform (Figure 2). A robotic safety harness gantry allows participants to move freely on the platform, but prevents falls if they are unable to regain balance. An accelerometer (Series 7523A, Dynamic Transducers and Systems, Chatsworth, California, USA) placed on the platform records platform acceleration. Eight high-resolution video cameras will be positioned around the room to collect markerless 3-dimensional kinematic data (Theia Markerless, Inc., Kingston, ON). Four force plates on the platform (1.5m x 1.5m, model BP11971197-2000, Advanced Mechanical Technology, Inc., Watertown, Massachusetts, USA) record forces and moments in 3 dimensions.

A baseline data collection session will be completed before the first training session. The forward multi-step threshold will be determined, using a staircase procedure.[27] Participants will initially experience a backward-directed platform perturbation (causing a forward 'fall') at 0.5 m/s^2 peak acceleration (intensity). Participants will be instructed to do whatever they need to do to recover balance but, if they need to take a step, to minimize the number of steps taken. If participants do not step or only take a single step to recover balance, the intensity will be increased in 0.5 m/s^2 increments until the participant needs to execute a multi-step reaction to recover balance. The intensity will then be reduced in 0.25 m/s^2 increments until the participant only takes one step (first reversal). The intensity will be increased, and then decreased, in 0.25 m/s^2 increments, reversing when the participant's step response changes (i.e., single- or multi-step reaction) until there are four reversals (Figure 3). The multi-step threshold is the highest perturbation magnitude where the participant can recover balance with a single step. The multi-step threshold should be around 2-3 m/s^2, which will take approximately 10-16 trials to determine.[27]

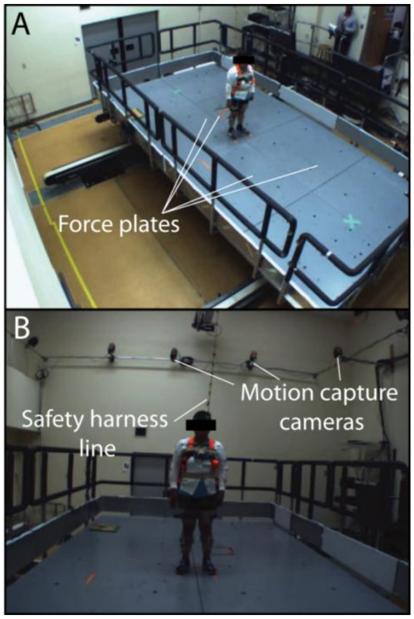

Figure 2: FallsLab motion platform and apparatus.

Participants assigned to the RBT groups will experience 36 multi-directional (left-, right-, and forward-fall) perturbations in each training session, presented in an unpredictable sequence. Participants assigned to **high-intensity RBT** will experience perturbations at 150% of the multi-step threshold; for example, for a multi-step threshold of 2 m/s^2 the high intensity will be 3 m/s^2.

Participants assigned to the **moderate-intensity RBT** group will experience perturbations at the multi-step threshold. If participants respond to the perturbations with a single step (high intensity group) or no step (moderate intensity group) in >50% of trials, the intensity will be increased by 25% in the subsequent training session. Participants in the **walking control** group will complete 36 unperturbed walking trials on the FallsLab platform in each training session.

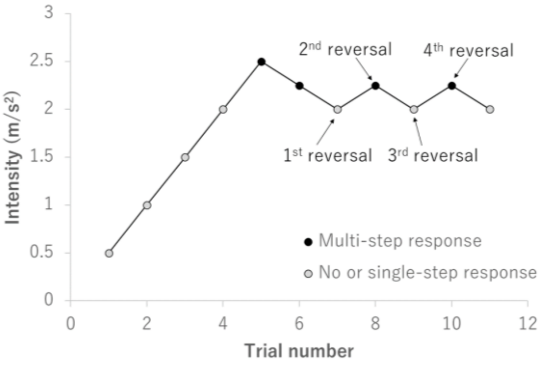

Figure 3: Example of the staircase procedure to determine the multi-step threshold. The first multi-step reaction is observed at 2.5m/s, at which point the intensity (peak acceleration) is reduced until a single step is observed. The perturbations are increased and decreased in $0.25m/s^2$ increments until there are four reversals of the 'staircase'.

2.3 Group allocation and blinding

Participants will be assigned using blocked (block size 6 or 9) randomization (computer-generated random sequence), stratified by sex to one of the three training groups. Participants cannot be blinded to group allocation for exercise trials. The primary outcome will be obtained during the training sessions and will be scored by a blinded adjudicator from raw data (videos). Secondary outcomes will be obtained by a blinded research assistant. To maintain allocation concealment, group allocation will be performed centrally by the principal investigator, who will not be involved in recruiting.

2.4 Participants

Community-dwelling men and women (>20 years old) with chronic stroke (>6 months post-stroke) will be recruited. Participants will be excluded if they:

- Are unable to stand independently without upper-limb support for >30 seconds and/or walk independently (without a gait aid) for ≥10 metres;
- Have another neurological condition that could affect balance control (e.g., Parkinson's disease);
- Have cognitive impairment (Montreal Cognitive Assessment[28] score <26[29]), or severe language or communication difficulties affecting understanding instructions;
- Have contraindications to RBT,[30] such as osteoporosis, activity restrictions due to cardiac event/surgery, or severe spasticity in the lower extremity; and/or
- Are currently attending in- or out-patient physiotherapy or supervised exercise.

Several strategies will be used to identify participants: 1) placing advertisements in the community (e.g., in health centres, local newspapers, or online; Appendix A); 2) requesting referrals from existing stroke rehabilitation programs at Toronto Rehab; and 3) Epic. For individuals responding to advertisements, the volunteer will be invited to contact the research team. For patients referred by healthcare providers at Toronto Rehab, initial contact may be made by a member of the research team (with the volunteer's consent) or the volunteer may choose to contact the research team. The healthcare provider may provide these patients with a study brochure (Appendix B) to give them some information about the study and help them to decide if they want to hear more. We will follow UHN guidance regarding identifying potential participants using Epic. To identify patients who are about to be discharged from rehabilitation and may be interested in participating in research, members of the research team will view the stroke in-patient and out-patient rehab program treatment schedules for patients that have indicated "Yes" to the "RSH (research) OK to contact" field in Epic. To identify patients with more chronic strokes, we will also submit a Data and Reporting Request for identifiers for UHN patients with a history of stroke that have indicated "Yes" to the "RSH (research) OK to contact" field in Epic. We will review patients' charts to pre-screen them using the criteria above. Eligible

patients will be contacted by the research team using the patients' preferred contact method, as indicated in Epic (Appendix L).

When initial contact is made, a member of the research team (DJ, CD, TNM) will explain the study and provide volunteers with the study information sheet and consent form (Appendix C). The informed consent checklist will be completed during this initial conversation to document the consent-related discussion between research staff and the patient. Patients may discuss the study with their friends, family members, or healthcare providers. Patients may take as long as necessary to decide if they wish to participate in the study; however, if a patient has not decided by one month after the initial discussion, we will assume they have declined participation. After participants provide consent, the eligibility checklist (Appendix D) will be completed to confirm eligibility. The hospital chart, members of the patient's healthcare team at the Toronto Rehabilitation Institute, and the participant will be consulted to confirm eligibility.

Participants will be informed during the consent process that they are free to withdraw from the study at any time point, without consequence. Participants will may also be withdrawn from the study due to changes in their health status (see Section 3.3.2).

To help to alleviate barriers to participating we will reimburse participants for travel expenses, and participants will receive a modest honorarium upon study completion.

2.5 Outcome measures

Outcome assessment will be repeated one week after the end of the training program to assess retention of training effects. Falls will be assessed for 12 months post-training.

2.8.1 Primary outcome: balance reactions to an untrained perturbation

Responses to backward-fall perturbations will be assessed at the end of each session, and at the one-week retention time-point. An untrained perturbation (i.e., backward-fall perturbation) will be used to test transfer of learning to a novel context.[25,26] To prevent participants from pre-planning their responses, the backward-fall perturbations will be completed in block of trials with 6 low-magnitude forward-fall and lateral perturbations (25-50% of participants' multi-step threshold), presented in an unpredictable sequence. Improved reactive stepping with practice of forward-fall perturbations transfers to reactions to backward-fall perturbations in people with chronic stroke.[31] Participants will experience 3 forward-directed platform translations, evoking a backward loss of balance, at the multi-step threshold. Because backward falls are more challenging than forward or lateral falls,[27,32] a backward-fall perturbation at the forward-fall multi-step threshold should evoke a multi-step reaction, at least prior to training. Testing at each participant's multi-step threshold ensures that participants will initially experience challenges responding to the perturbation and there is room for improvement with training. The primary outcome will be number of steps taken to recover balance. Extra steps are required when the first step is ineffective for regaining balance; less effective initial steps require more extra steps.[22,23] Multi-step reactions are associated with increased risk of falls in daily life,[33] and people reduce the number of steps taken to recover balance after RBT.[11,13,31] Therefore, number of steps taken to recover balance is a global indicator of effectiveness of balance reactions.[23,24]

We will calculate the learning rate for each participant by fitting an exponential function, $a + b\left(e^{-\frac{t}{x}}\right)$, to their data (average number of steps taken at each assessment time point),[34] where a and b are constants, t is the assessment time point, and x is the learning rate. Figure 4 illustrates possible results for the three groups, using three different learning curves (x=0.4, 2 and 10 for the high-intensity, moderate-intensity, and walking control groups, respectively).

2.8.2 Secondary outcomes: adverse outcomes, mechanisms underlying improved reactive stepping, functional balance, and falls efficacy

Adverse outcomes will be monitored, recorded, and reported according to the ExHaRM protocol.[35] The physiotherapist will observe and monitor participants for adverse outcomes during the sessions (e.g., injuries, pain, expressions of fear/anxiety). Participants will also be prompted to report any adverse outcomes they experienced after the session at the subsequent session by asking "since our last session, have you experienced any harmful or undesirable outcomes, whether or not you think they were caused by exercise?".[35] Adverse outcomes will be documented in Epic using the Common Terminology Criteria for Adverse Events (version 5.0; CTCAE).[36] Psychological adverse outcomes (i.e., fear and anxiety related to the perturbations) are not included within the CTCAE, and will documented separately. Documentation will include a narrative description of the adverse outcome, adverse outcome category, severity, whether the adverse outcome is attributed to the study intervention/procedures, and impact of the adverse outcome on the study. The physiotherapist will ensure that participants receive appropriate care when adverse outcomes require immediate attention (e.g., injuries); depending on the severity of these adverse events, the participant may be withdrawn from the study. The physiotherapist may modify the intervention in response to less severe adverse outcomes (e.g., reducing the number of perturbations due to fatigue, mild pain, or fear/anxiety).

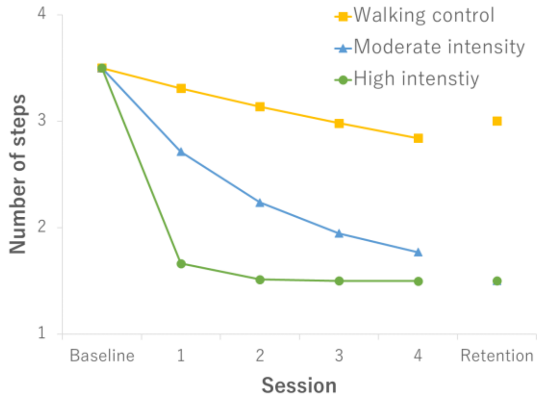

Figure 4: Illustration of possible learning curves for the three groups. Learning curves were created using expected pre-training average of 3-4 steps, and post-training average of 1-2 steps for the trained groups.

The initial step reaction may be ineffective for several reasons (e.g., too short or slow),[22] necessitating additional steps to recover balance. To explore the mechanisms underlying improved reactive stepping with practice, we will also obtain the following outcomes from the force plates and motion capture system during the backward-fall perturbations: step initiation time (from perturbation onset to foot off), step execution time (from foot off to foot contact of the first step), step length (difference in foot position from foot off to foot contact), braking impulse (antero-posterior shear force generated over time on step contact),[37,38] and mechanical margin of stability at foot contact.[39] Margin of stability is the distance between the extrapolated centre of mass (incorporating centre of mass position and velocity) and the edge of the base of support. Low values indicate that the centre of mass is close to and/or moving quickly towards the edge of the base of support and, therefore, that the person is close to falling (i.e., less stable).

Functional balance will be assessed using the mini-Balance Evaluation Systems test (mini-BEST),[40] which is a 14-item observational rating scale that assesses balance systems, including reactive balance control.[40] The mini-BEST has good inter- (ICC=0.96) and intra-rater reliability (ICC=0.97) in chronic stroke.[41] The mini-BEST reactive balance control sub-scale will provide further support for transfer of training effects to untrained contexts.

We will use additional recommended secondary outcomes from the Core Outcome Set for Evaluating Mixed-Diagnosis Falls Prevention Interventions;[42] that is: 1) fall incidence; 2) injurious fall incidence; 3) quality of life (5-level EuroQoL health status measure; EQ-5D-5L[43,44]); 4) falls self-efficacy (Falls Efficacy Scale International; FES-I[45]); 5) fear of falling; and 6) activity curtailment due to fear of falling. These outcomes, except for the FES-I, are collected to harmonize outcomes with other fall prevention studies and will not be analyzed to address a specific study objective or hypotheses. To assess fall incidence and injurious fall incidence, participants will be asked to report falls ("an event that results in a person coming to rest unintentionally on the ground or other lower level"[46]) for 12 months post-training. Participants will be provided with stamped, addressed postcards

to mail to the research team every month for 1 year post-training. Postcards will contain a calendar, on which participants will record falls. The blinded research assistant will call participants who do not return the postcard to determine if any falls occurred. The research assistant will also contact participants reporting a fall to complete a short questionnaire determining the cause and consequences of the fall (e.g., injuries; Appendix E). This method is considered the 'gold standard' for fall reporting[47] and has been successfully used by our team.[12,48,49] The 5-level EuroQoL health status measure (EQ-5D-5L)[43,44] asks participants to rate their health in five dimensions (mobility, self-care, usual activities, pain/discomfort, and anxiety/depression) on a five-point scale and their overall health state on a scale from 0-100.[43] The EQ-5D-5L has good-to-excellent test-retest reliability across several populations (ICC: 0.69-0.93).[50] The FES-I asks participants to rate their concern about falling while performing 16 everyday tasks.[45] The FES-I shows excellent test-retest reliability (ICC=0.97) in people with chronic stroke.[51] Fear of falling and activity curtailment due to fear of falling will be assessed with two questions ("are you afraid of falling?" and "do you think fear of falling has made you cut down on any activities that you used to do?").[52]

The mini-BEST, EQ-5D-5L, FES-I, fear of falling, and activity curtailment due to fear of falling will be assessed pre- and post-training. Additionally, the EQ-5D-5L, FES-I, fear of falling, and activity curtailment due to fear of falling will be repeated via telephone at the end of the 1-year falls monitoring period (Figure 1).

2.8.3 Cohort descriptors

The following information will be recorded at study enrolment to characterize the cohort: age, sex, gender identity, height, weight, time since stroke, lesion location, pre-morbid medical history, prescription medications, the National Institutes of Health Stroke Scale (stroke severity),[53] Chedoke-McMaster Stroke Assessment[54] foot and leg scores (motor impairment), and the Nielsen gender questionnaire.[55] Cohort descriptors will be obtained directly from participants, or from Epic (Appendix F).

2.6 Sample size justification

Our primary outcome is learning rate. The sample size for each group was estimated as λ/Δ,[56] where λ was obtained from tables for 3 groups using probability of a Type 1 error (α) of 0.05, probability of a Type II error (β) of 0.2; therefore, λ of 9.64 was used. Additionally:

$$\Delta = \frac{1}{\sigma^2}\sum_{i=1}^{k}(\mu_i - \bar{\mu})^2$$

where σ is the standard deviation, k is the number of groups (3), μ_i is the mean for each groups and $\bar{\mu}$ is the mean for all groups combined. From pilot data, we estimate a standard deviation of 9.7 for the learning rate. People with stroke typically require 3-4 steps to regain stability following the perturbation magnitudes used in the proposed study.[13,57] Due to the mechanical challenges of the perturbation, at minimum 1 step is required to respond to the perturbation (i.e., young healthy adults would take 1 step to respond to the perturbation). Therefore, an improvement from 3-4 steps pre-training to 1-2 steps post-training is considered clinically meaningful, and a much larger effect size would likely not be possible. From previous studies[13] an improvement from taking, on average, 3-4 steps pre-training to 1-2 steps post-training is feasible. Simulating learning rates between these data points (Figure 5), we expect that learning rates of approximately 0.4 (μ_1), 2 (μ_2) and 10 (μ_3) for the high-intensity, moderate-intensity, and walking control groups, respectively, would show meaningful differences between the three groups. Therefore, Δ was calculated as 0.56, giving a target sample size of 17 per group or 51 participants total. To account for a ~20% rate of withdrawal,[11,12,58] we will aim to recruit 21 participants per group, or 63 participants total. Enrolment will stop when 51 participants have completed the retention test, or 63 participants have been enrolled, whichever happens first.

2.7 Analyses

One-way analysis of covariance (ANCOVA), with sex as a covariate, will be used to compare learning rates between the 3 groups. ANCOVA, controlling for the pre-training value, will be used to compare the following outcomes between the 3 groups at the post-training and retention time points: average number of steps, step initiation time, step execution time, step length, braking impulse, margin of stability, mini-BEST, and FES-I. Pre-planned contrasts will be used to compare outcomes between the high- and moderate-intensity groups, and between the two RBT groups combined and the walking control group. Alpha will be 0.05 for the primary outcome (learning rates), and adjusted for multiple comparisons using the Holm-Bonferroni method[59] for secondary outcomes. Falls and quality of life data will be reported without inference so that these data can be used in meta-analyses and/or as pilot data for future studies.

3. TRIAL MANAGEMENT

3.1 Arrangements for day-to-day trial management

The principal investigator (PI) will be responsible for study oversight. A graduate student (TNM), will be responsible for ensuring day-to-day conduct of the trial, under direction of the PI. A blinded research assistant (DJ) will assist with trial administration, recruiting, and data collection. A physiotherapist (CJD) with expertise in RBT will oversee the training sessions.

3.2 Risk management

3.2.1 Balance testing and training

With appropriate screening, the risks of study procedures and interventions are minimal. RBT can be completed by people with stroke with few adverse events.[12,49,60,61] Mild adverse events related to RBT included delayed-onset muscle soreness, fatigue, or exacerbation of joint pain (11%, 7%, and 32% of participants, respectively),[12] which did not require medical attention, but resulted in modifying the intervention until they resolved (typically by the following session). Of note, the frequency and severity of adverse events were similar for the RBT group and control group, who completed more 'traditional' balance training.[12] When more frequent or severe adverse outcomes are reported in RBT, this is typically due to use of very high intensity perturbations (where participants fall into the safety harness) or increased reports of anxiety related to the perturbations. We will avoid using these very high intensity perturbations to reduce the risk of more severe adverse outcomes. Participants will wear a safety harness attached securely overhead to prevent falling during training, and training sessions will be overseen by a licenced physiotherapist with experience conducting RBT. Additional actions may be taken to prevent risk of injury for specific participants (e.g., wearing an ankle brace for participants with ankle instability).

As the assessment and intervention includes tasks that are challenging to balance control, there is a small risk that participants will lose their balance and fall. Appropriate precautions will be taken to ensure patient safety during these tasks. Interventions will be administered by a trained and licensed physiotherapist. Assessments will be completed by a trained research assistant with a health sciences background. A safety harness attached to a secure point overhead will be worn for all postural perturbations to prevent a fall to the floor if the individual fails to regain stability. We have administered tens of thousands of postural perturbations to over 500 people with varying balance abilities in previous research studies and clinical activities and no participant suffered an injury as a result of an induced postural perturbation. However, even if the participant is caught by the safety harness, there is a very small chance that participants will suffer a physical injury (e.g., sprain or bruise). In the event of a minor physical injury, the physiotherapist will provide first aid, will advise the participant regarding follow-up with a medical professional (e.g., family doctor) and home treatment (e.g., rest, ice, compression, elevation), and will follow-up with the participant after a day or two.

3.2.2 Health status

Participants will be withdrawn if their health changes such that they would no longer be eligible for the study (i.e., one of the exclusion criteria applies to them). Changes in health status may be communicated to the research team via Epic or directly from the participant.

3.2.3 Privacy and confidentiality

Personal information is any information that could identify participants. If participants agree to join this study, the following personal information will only be accessible to the research team, for contact purposes:

- name
- telephone number and/or e-mail addressed (based on participant preference)
- address
- telephone number of alternative contact

A number of steps will be taken to ensure protection of personal health information. All information collected during this study, including the participant's personal information, will be kept confidential and will not be shared with anyone outside the study unless required by law. Electronic data will be stored on secure UHN servers for 10 years. After 10 years the data will be deleted from the UHN servers. Electronic files containing patient names and contact information will be password protected, and will be stored separately from study data. Hard copies of files containing de-identified data will be stored in locked cabinets and/or in offices that are locked when not occupied. Consent forms will be stored in locked cabinets/offices separately from other data. Only those individuals who require access to the data for the purpose of this study will be provided with the password to the file containing identifiers and/or the keys to the locked cabinet/office.

Video recordings of participants during the balance tests will remain within the study team, unless an optional media consent form was completed by the participant (Appendix G); in which, members of the research team could show the images only to limited academic audiences (e.g., at scientific/clinical conferences or in lectures to university students). Presenting data, including images from the study, to other researchers and students helps to share our study findings with people who can use the findings of the study. Alternatively, the images could be shown to the general public, including being posted on public websites, like YouTube. Sharing information about our study publicly helps more people to learn about our study findings. In this case, the images may be easily copied, downloaded and reused by people outside of the research team.

3.3 Follow-up with participants

Participants will be asked to complete a Research Volunteer Satisfaction survey (Appendices H & I) when they complete the study. The purpose of the survey is for us to learn more about the experiences of the research participants in this study. This information may be used in the design of future studies. Participants may choose to complete the survey anonymously, but may also wish to provide their contact information for follow-up with the principal investigator. Completed surveys will be stored separately from participants' study file. Participants will receive a letter of appreciation at the end of the study (Appendix J).

4. IMPACT

4.1 Significance

A high rate of falling is a common after stroke, and fall risk is highest in the first months post-discharge from rehabilitation.[62] RBT is a novel type of exercise that aims to improve reactive balance control,

rather than ‘traditional’ balance training, which focuses on maintaining stability during voluntary movement. Time in stroke rehabilitation is limited, and physiotherapists report lack of time is a barrier to implementing RBT.[17] The results of the proposed study will inform clinicians about the optimal RBT training parameters to improve reactive balance control and reduce risk of falling post-stroke. We expect to find the high intensity RBT leads to faster improvements in reactive stepping ability than moderate intensity RBT. This finding would suggest that clients can achieve the same benefits from a short duration of high-intensity RBT as for a longer duration of moderate-intensity training. Therefore, clinicians with limited time in rehabilitation can use high-intensity RBT to improve their clients’ reactive balance control in a short amount of time, without sacrificing time spent on other important rehabilitation goals. Alternatively, if a client is unable to tolerate high-intensity perturbations, then the study findings would suggest that a longer duration of RBT would be needed to improve reactive balance control.

4.2 Knowledge translation

Study results will be shared with the academic community via publication in peer-reviewed journals and presentations at conferences. We will share results directly with physiotherapists through interactive workshops (e.g., at the Canadian Physiotherapy Association meeting). The results of the trial will be incorporated into our RBT toolkit.[30]

5. LIST OF APPENDICES

Appendix	Description	Version date
A	Advertisement poster	15 July 2024
B	Study brochure	18 April 2024
C	Consent form	29 July 2024
D	Eligibility checklist	12 April 2024
E	Falls reporting questionnaire	18 April 2024
F	Chart review form	8 May 2024
G	Optional media consent form	18 April 2024
H	Volunteer satisfaction survey	18 April 2024
I	Volunteer Satisfaction survey cover letter	8 May 2024
J	Letter of appreciation	4 July 2024
K	Falls reporting calendar	4 July 2024
L	Initial contact template (Epic)	4 July 2024

REFERENCES

1. Greenlund KJ, Giles WH, Keenan NL, Croft JB, Mensah GA. Physician advice, patient actions, and health-related quality of life in secondary prevention of stroke through diet and exercise. *Stroke.* 2002;33(2):565-570.
2. Andersson ÅG, Kamwendo K, Apperlros P. Fear of falling in stroke patients: relationship with previous falls and functional characteristics. *Int J Rehabil Res.* 2008;31:261-264.
3. Mansfield A, Inness EL, McIlroy WE. Stroke. In: Day BL, Lord SR, eds. *Handbook of Clinical Neurology: Balance, Gait, and Falls.* Vol 159. San Diego: Elsevier BV; 2018:205-228.
4. Schmid AA, Van Puymbroeck M, Altenburger PA, et al. Balance and balance self-efficacy are associated with activity and participation after stroke: a cross-sectional study in people with chronic stroke. *Arch Phys Med Rehabil.* 2012;93:1101-1107.
5. Mackintosh SFH, Hill K, Dodd KJ, Goldie P. Falls and injury prevention should be part of every stroke rehabilitation plan. *Clin Rehabil.* 2005;19:441-451.
6. Sherrington C, Fairhall NJ, Wallbank GK, et al. Exercise for preventing falls in older people living in the community. *Cochrane Database Syst Rev.* 2019;2019(1):CD012424.
7. Sibley KM, Thomas SM, Veroniki AA, et al. Comparative effectiveness of exercise interventions for preventing falls in older adults: a secondary analysis of a systematic review with network meta-analysis. *Exp Gerontol.* 2020;143(11):111151.
8. Denissen S, Staring W, Kunkel D, et al. Interventions for preventing falls in people after stroke. *Cochrane Database Syst Rev.* 2019;10(10):CD008728.
9. Maki BE, McIlroy WE. Postural control in the older adult. *Clin Geriatr Med.* 1996;12(4):635-658.
10. Mansfield A, Peters AL, Liu BA, Maki BE. A perturbation-based balance training program for older adults: study protocol for a randomised controlled trial. *BMC Geriatr.* 2007;7(1):12.
11. Mansfield A, Peters AL, Liu BA, Maki BE. Effect of a perturbation-based balance-training program on compensatory stepping and grasping reactions in older adults: a randomized controlled trial. *Phys Ther.* 2010;90(4):476-491.
12. Mansfield A, Aqui A, Danells CJ, et al. Does perturbation-based balance training prevent falls among individuals with chronic stroke? A randomised controlled trial. *BMJ Open.* 2018;8:e021510.
13. Schinkel-Ivy A, Huntley AH, Aqui A, Mansfield A. Does perturbation-based balance training improve control of reactive stepping in individuals with chronic stroke. *J Stroke Cerebrovasc Dis.* 2019;28(4):935-943.
14. Schinkel-Ivy A, Huntley AH, Danells CJ, Inness EL, Mansfield A. Improvements in balance reaction impairments following reactive balance training in individuals with sub-acute stroke. *Top Stroke Rehabil.* 2020;27(4):262-271.
15. Mansfield A, Inness EL, Komar J, et al. Training rapid stepping responses in an individual with stroke. *Phys Ther.* 2011;91(6):958-969.
16. Devasahayam AJ, Farwell K, Lim B, et al. The effect of reactive balance training on falls in daily life: an updated systematic review and meta-analysis. *Phys Ther.* 2023;103(1):pzac154.
17. Mansfield A, Danells CJ, Inness EL, Musselman KE, Salbach NM. A survey of Canadian healthcare professionals' practices regarding reactive balance training. *Physiother Theory Pract.* 2021;37(7):787-800.
18. Jagroop D, Houvardas S, Danells CJ, et al. Rehabilitation clinicians' perspectives of reactive balance training. *Disabil Rehabil.* 2022;44(25):7967-7973.
19. Bray NW, Smart RR, Jakobi JM, Jones GR. Exercise prescription to reverse frailty. *Appl Physiol Nutr Metab.* 2016;41:1112-1116.

20. MacInnis MJ, Gibala MJ. Physiological adaptations to interval training and the role of exercise intensity. *J Physiol.* 2017;595(9):2915-2930.

21. Wewege M, van den Berg R, Ward RE, Keech A. The effects of high-intensity interval training vs moderate-intensity continuous training on body composition in overweight and obsese adults: a systematic review and meta-analysis. *Obes Rev.* 2017;18(6):635-646.

22. Maki BE, McIlroy WE. The role of limb movements in maintaining upright stance: the "change-in-support" strategy. *Phys Ther.* 1997;77:488-507.

23. Wolfson LI, Whipple R, Amerman P, Kleinberg A. Stressing the postural response: a quantitative method for testing balance. *J Am Geriatr Soc.* 1986;34:845-850.

24. Jacobs JV, Horak FB, Tran VK, Nutt JG. An alternative clinical postural stability test for patients with Parkinson's disease. *J Neurol.* 2006;253:1404-1413.

25. Gregor S, Saumur TM, Crosby LD, Powers J, Patterson KK. Study paradigms and principles investigated in motor learning research after stroke: a scoping review. *Arch Rehabil Res Clin Transl.* 2021;3(2):100111.

26. Schmidt RA, Bjork RA. New conceptualizations of practice: common principles in three paradigms suggest new concepts for training. *Psychol Sci.* 1992;3(4):207-217.

27. de Kam D, Roelofs JMB, Bruijnes AKBD, Geurts ACH, Weerdesteyn V. The next step in understanding impaired reactive balance control in people with stroke: the role of defective early automatic postural responses. *Neurorehabil Neural Repair.* 2017;31(8):708-716.

28. Nasreddine ZS, Phillips NA, Bédirian V, et al. The Montreal cognitive assessment (MoCA): a brief screening tool for mild cognitive impairment. *J Am Geriatr Soc.* 2005;53:695-699.

29. Gagnon G, Hansen KT, Woolmore-Goodwin S, et al. Correcting the MoCA for education: effect on sensitivity. *Can J Neurol Sci.* 2013;40:678-683.

30. Mansfield A, Inness EL, Danells CJ, et al. Implementing reactive balance training in rehabilitation practice: a guide for healthcare professionals. 2021.

31. Dusane S, Wang E, Bhatt T. Transfer of reactive balance adaptation from stance-slip perturbation to stance-trip perturbation in chronic stroke survivors. *Restor Neurol Neurosci.* 2019;37:469-482.

32. McIlroy WE, Maki BE. Age-related changes in compensatory stepping in response to unpredictable perturbations. *J Gerontol A Biol Sci Med Sci.* 1996;51A(6):M289-296.

33. Carty CP, Cronin NJ, Nicholson D, et al. Reactive stepping behaviour in response to forward loss of balance predicts future falls in community-dwelling older adults. *Age Ageing.* 2015;44(1):109-115.

34. van Beers RJ. How does our motor system determine its learning rate. *PLoS ONE.* 2012;7(11):e47373.

35. Spence RR, Sandler CX, Jones TL, Mcdonald N, Dunn RM, Hayes SC. Practical suggestions for harms reporting in exercise oncology: the Exercise Harms Reporting Method (ExHaRM). *BMJ Open.* 2022;12:e067998.

36. National Cancer Institute. Common Terminolgy Criteria for Adverse Events (CTCAE). 2021; https://ctep.cancer.gov/protocoldevelopment/electronic_applications/ctc.htm. Accessed 20 July, 2023.

37. Hsiao-Wecksler ET. Biomechanical and age-related differences in balance recovery using the tether-release method. *J Electromyogr Kinesiol.* 2008;18(2):179-187.

38. King GW, Luchies CW, Stylianou AP, Schiffman JM, Thelen DG. Effects of step length on stepping response to arrest a forward fall. *Gait Posture.* 2005;22(3):219-224.

39. Hof AL, Gazendam MGJ, Sinke WE. The condition for dynamic stability. *J Biomech.* 2005;38:1-8.

40. Frachignoni F, Horak F, Godi M, Nardone A, Giordani A. Using psychometric techniques to improve the balance evaulation systems test: the mini-BES test. *J Rehabil Med.* 2010;42(4):323-331.

41. Tsang CSL, Liao L-R, Chung RCK, Pang MYC. Psychometric properties of the mini-Balance Evaluation Systems test (mini-BES test) in community-dwelling individuals with chronic stroke. *Phys Ther.* 2013;93(8):1102-1115.

42. O'Malley N, Coote S, Clifford AM. Protocol for the development of a core outcome set for evaluating mixed-diagnosis falls prevention interventions for people with multiple sclerosis, Parkinson's disease and stroke. *HRB Open Res.* 2022;4:123.

43. Rabin R, de Charro F. EQ-5D: a measure of health status from the EuroQol Group. *Ann Med.* 2001;33(5):337-343.

44. Herdman M, Gudex C, Lloyd A, et al. Development and preliminary testing of the new five-level version of EQ-5D (EQ-5D-5L). *Qual Life Res.* 2011;20(10):1727-1736.

45. Yardley L, Beyer N, Hauer K, Kempen G, Piot-Ziegler C, Todd C. Development and initial validation of the Falls Efficacy Scale-International (FES-I). *Age Ageing.* 2005;34:614-619.

46. Hyndman D, Ashburn A, Stack E. Fall events among people with stroke living in the community: circumstances of falls and characteristics of fallers. *Arch Phys Med Rehabil.* 2002;83:165-170.

47. Myers AH, Baker SP, van Natta ML, Abbey H, Robinson EG. Risk factors associated with falls and injuries among elderly institutionalized persons. *Am J Epidemiol.* 1991;133:1179-1190.

48. Mansfield A, Wong JS, McIlroy WE, et al. Do measures of reactive balance control predict falls in people with stroke returning to the community? *Physiotherapy.* 2015;101(4):373-380.

49. Mansfield A, Schinkel-Ivy A, Danells CJ, et al. Does perturbation training prevent falls after discharge from stroke rehabilitation? A prospective cohort study with historical control. *J Stroke Cerebrovasc Dis.* 2017;26(10):2174-2180.

50. Buchholz I, Janssen MF, Kohlmann T, Feng Y-S. A systematic review of studies comparing the measurement properties of the three-level and five-level versions of the EQ-5D. *Pharmacoeconomics.* 2018;36(6):645-661.

51. Hellström K, Lindmark B. Fear of falling in patients with stroke: a reliability study. *Clin Rehabil.* 1999;13:509-517.

52. Perez-Jara J, Walker D, Heslop P, Robinson S. Measuring fear of falling and its effect on quality of life and activity. *Rev Clin Gerontol.* 2010.

53. Goldstein LB, Bertels C, Davis JN. Interrater reliability of the NIH Stroke Scale. *Arch Neurol.* 1989;46(6):660-662.

54. Gowland C, Stratford P, Ward M, et al. Measuring physical impairment and disability with the Chedoke-McMaster Stroke Assessment. *Stroke.* 1993;24:58-63.

55. Nielsen MW, Stefanick ML, Peragine D, et al. Gender-related variables for health research. *Biol Sex Differ.* 2021;12:23.

56. Chow S-C, Shao J, Wang H. *Sample Size Calculations in Clinical Research.* 3rd ed: CRC Press; 2018.

57. Schinkel-Ivy A, Aqui A, Danells CJ, Mansfield A. Characterization of reactions to laterally directed perturbations in people with chronic stroke. *Phys Ther.* 2018;98(7):585-594.

58. Marigold DS, Eng JJ, Dawson AS, Inglis JT, Harris JE, Gylfadóttir S. Exercise leads to faster postural reflexes, improved balance and mobility, and fewer falls in older persons with chronic stroke. *J Am Geriatr Soc.* 2005;53:416-423.

59. Holm S. A simple sequentially rejective multiple test procedure. *Scand J Statist.* 1979;6:65-70.

60. Handelzalts S, Kenner-Furman M, Gray G, Soroker N, Shani G, Melzer I. Effects of perturbation-based balance training in subacute persons with stroke: a randomized controlled trial. *Neurorehabil Neural Repair.* 2019;33(3):213-224.

61. Bhatt T, Dusane S, Patel P. Does severity of motor impairment affect reactive adaptation and fall-risk in chronic stroke survivors? *J Neuroeng Rehabil.* 2019;16(1).

62. Forster A, Young J. Incidence and consequences of falls due to stroke: a systematic inquiry. *BMJ.* 1995;311:83-86.
