# Supplementary material for: Investigating the optimal reactive balance training intensity in people with chronic stroke: Study protocol for a randomized control trial
Source: PLoS One. 2025 Sep 9;20(9):e0327937. doi: 10.1371/journal.pone.0327937 (PMC12419664; doi:10.1371/journal.pone.0327937)
Supplement: S3 File — (PDF) [file pone.0327937.s003.pdf]

Informed consent form for participation in a research study

Study title: Optimal intensity of reactive balance training post-stroke: a randomized controlled trial

Study researcher: Avril Mansfield, R.Kin, PhD; KITE-Toronto Rehabilitation Institute; 416-597-3422 ext 7831

Funder: This study is funded by the Heart and Stroke Foundation of Canada

INTRODUCTION
You are being invited to take part in a clinical trial. You are invited to participate in this clinical trial because you have had a stroke. This consent form provides you with information to help you make an informed choice. Please read this document carefully and ask any questions you may have. All your questions should be answered to your satisfaction before you decide whether to participate in this research study. You may find it helpful to discuss it with your friends and family.

Please take your time in making this decision.

Taking part in this study is voluntary. You have the option to not participate at all, or you may choose to leave the study at any time. Whatever you choose, it will not affect the usual medical care that you receive outside of the study.

IS THERE A CONFLICT OF INTEREST?
The University Health Network is receiving financial payment from the Heart and Stroke Foundation to cover the cost of conducting this study.

WHAT IS THE BACKGROUND INFORMATION FOR THIS STUDY?
People who have had a stroke tend to have ‘poor’ balance and are more likely to fall than those who have not had a stroke. A new type of exercise, called ‘reactive balance training’, may help reduce fall rates after stroke. Some studies suggest that people can get more benefit from more challenging reactive balance training, but we do not know if this is true for people with stroke.

WHY IS THIS STUDY BEING DONE?
The purpose of this study is to find out if highly challenging reactive balance training improves balance more than more moderately challenging reactive balance training or walking training.

WHAT OTHER CHOICES ARE THERE?

The healthcare system currently does not provide rehabilitation to people with stroke after a certain amount of time.

- If you think you should improve your balance or are concerned about falling, you can hire a private physiotherapist, kinesiologist, or fitness instructor who can help you.
- You may also seek out community exercise programs that could benefit you. Our study staff are aware of some of these programs and help to connect you with a program.

HOW MANY PEOPLE WILL TAKE PART IN THIS STUDY?

It is anticipated that 63 people will take part in this study. The study should take about 3 years to complete and the results should be known by June 2027.

WHAT WILL HAPPEN DURING THIS STUDY?

If you decide to participate then you will be "randomized" into one of the groups described below. Randomization means that you are put into a group by chance. There is no way to predict which group you will be assigned to. You will have a one in three chance of being placed in any group. Neither you, the study staff, nor the researchers can choose what group you will be in.

- Group 1 participants will be asked to do highly challenging reactive balance training.
- Group 2 participants will be asked to do moderately challenging reactive balance training.
- Group 3 participants will be asked to do walking training and low-to-moderately challenging reactive balance training.

You will know if you are in Group 3, but if you are assigned to one of the reactive balance training groups, you will not be told if you are in Group 1 or Group 2.

When you are finished with your participation in the study and would like to know what study group you were in, please let the study team know.

WHAT IS THE STUDY INTERVENTION?

- Group 1 will do 4 sessions of 1-hour long highly challenging reactive balance training. Participants will stand on a platform that will move suddenly to disturb their balance. Participants will need to react quickly to regain their balance after the platform moves. Participants will practice responding to different platform movements during the training session.
- Group 2 will do the same training as participants in Group 1, but the training will be only moderately challenging. This means that the speed of the platform movements will be slower for Group 2 than Group 1.
- Group 3 will do 4 sessions of 1-hour long walking training. Participants will walk across the platform, but the platform will not move to disturb their balance. Participants in Group 3 will also do some reactive balance training,

but the training will be of a low-to-moderate challenge. This means that, in general, the speed of the platform movements will be slower for Group 3 than for Group 1 or Group 2.
- All participants must complete the study intervention consecutively (4 days in a row).

WHAT ELSE DO I NEED TO KNOW ABOUT THE STUDY INTERVENTION?

If you report pain because of the intervention, the study physiotherapist may reduce the challenge of the training, or reduce the amount of training you do, until the pain resolves.

WHAT ARE THE STUDY PROCEDURES?

Study periods:

This study involves 3 periods: Screening, Balance testing & training, and Falls monitoring

- **Screening**: We will ask you some questions about you and your health history to make sure you are eligible to participate in the study. It will take about 10-15 minutes to ask these questions. If you were previously a patient at UHN, we will review your hospital chart to get some information about your stroke, your previous medical history, and your current prescription medications. We use this information to confirm that you are eligible for the study and to describe the type of people who have participated in the study. You do not need to do anything additional for the chart review.
- **Balance and functional testing**: If you are eligible to participate. You will be asked to complete two testing sessions: 1) the day before you start the intervention; and 2) the week after you finish the intervention. The first testing session will last about 1.5 hours, and the second testing session will last about 1 hour. You can take rest breaks as often as you need during the testing sessions. During these test sessions, we will ask you several questions and conduct several tests, described below.
- **Falls reporting**: After you do the balance training, you will be given a monthly calendar that you will fill out daily for one year.

Balance and functional testing procedures

- Stroke function tests (20 minutes, first visit only) - we will measure your height and weight. We will do some quick tests of your vision, memory, sense of touch, and arm and leg function. These tests tell us how your stroke has affected you. We use this information to describe the kind of people who participate in the study.

- Leg and foot recovery (10 minutes, first visit only) – we will ask you to do a few movements with your leg and foot that have been affected by the

stroke, such as bending the knee or wiggling the toes. We use this information to describe the kind of people who participate in the study.

- Questionnaire about you (10 minutes, first visit only) – we will ask you to complete a questionnaire on paper about your employment and caregiving responsibilities, social supports, risk taking, and experiences with discrimination. We use this information to describe the kind of people who participate in the study. You are free to choose not to answer any of the questions. You can take the questionnaire away with you and answer it at home if you like.

- Health and balance confidence questionnaire (10 minutes) - we will ask you to complete a questionnaire on paper about your feelings about your health and balance confidence. We would like to know if feelings about health and balance confidence improves after completing the training. You are free to choose not to answer any of the questions. You can take the questionnaire away with you and answer it at home if you like (first visit only).

- Balance test (15 minutes) - we will ask you to do several activities that challenge your balance and mobility, such as walking as quickly as you can, standing with your eyes closed, and recovering your balance once released from a leaning position. A research assistant will stand near you when you complete the tests to provide any assistance you might need. You may be asked to wear a brace around your ankle during these tests if your ankle seems to be unstable. The research assistant will rate how you perform on each test. We would like to know if your ability to perform these tests improves after completing the training.

- Balance reaction test (15 minutes) - we will test your balance reactions on a movable platform. During this test, you will wear a safety harness attached to an overhead beam. We will ask you to stand on the platform. At an unexpected time, the platform will move suddenly and stop to disturb your balance. We will gradually increase the speed of the platform movements until we find speed that is challenging enough that you need to take a few steps to recover. You may be asked to wear a brace around your ankle during these tests if your ankle seems to be unstable.

The balance reaction tests will be videotaped so that we can check how you performed the tests after you finish your appointment. The videotaping is mandatory for the study. The video images will include your face and body. Your face will not be blurred in the video images. Only study personnel will have access to your video images.

Falls reporting procedures

When you have finished the balance training, you will be given a monthly calendar that you will be asked to fill out daily. You will use this calendar to

record any falls or near falls that you experience. We will ask you to return the calendar to us monthly. If you experience a fall or a near fall, it is important that you get the medical care you may need. After your medical care is addressed, you will be asked to contact us via phone to answer some questions about the fall or near fall. You can answer these questions over the telephone. The questions include what you were doing when you fell, what you think caused the fall, and whether you have a fear of falling. The questions should take 15-30 minutes to answer.

If you do not return a calendar we will call you to remind you to return it. We will also call you at the end of this 12-month monitoring to ask you questions about your feelings about your health and balance confidence. These questions should take about 10 minutes to answer.

<u>Summary of tests and procedures</u>

Study period	Time	Visit	Approximate length
Screening			10-15 minutes
Balance and functional testing<br>• Stroke function tests<br>• Leg and foot recovery<br>• Questionnaire about you<br>• Health and balance confidence questionnaire<br>• Balance test<br>• Balance reaction test	Week 1	1	1.5 hours
Balance training	Week 1	2	1 hour
		3	1 hour
		4	1 hour
		5	1 hour
Balance and functional testing<br>• Health and balance confidence questionnaire<br>• Balance test<br>• Balance reaction test	Week 2	6	1 hour
Falls reporting	Weeks 3-54	-	-

<u>Optional use of video recording</u>

We may ask for your permission to show the videos to some people outside the study (e.g., for educational purposes). This is optional. Optional means you do not have to agree to this use of your video recording to participate in the study. You will be provided a separate consent form for this. We will not share the videos with anyone outside of the study without your permission.

<u>Optional volunteer satisfaction survey</u>

When you have completed the study, you will be asked to complete a research volunteer satisfaction survey by mail. The purpose of the survey is for us to learn

more about the experiences of the research participants in this study. This information may be used in the design of future studies. You will receive the questionnaire via the mail. Completing the survey is optional.

WHAT ARE THE RESPONSIBILITIES FOR STUDY PARTICIPANTS?
If you choose to participate in this study, you will be expected to:
- Tell the study staff about your current medical conditions.
- Tell the study staff about all prescription and non-prescription medications and supplements, including vitamins and herbals.
- If you need device to see, hear, or move better, like eye glasses, hearing aids, an arm sling or a brace around your ankle/knee, bring these to your study appointments.
- Tell the study staff if you are thinking about participating in another research study.
- Return the falls reporting calendars and any questionnaires that you take home to complete.

HOW LONG WILL PARTICIPANTS BE IN THE STUDY?
You will be in the study for 1 year total.

CAN PARTICIPANTS CHOOSE TO LEAVE THE STUDY?
You can choose to end your participation in this research (called withdrawal) at any time without having to provide a reason. If you choose to withdraw from the study, you are encouraged to contact the study staff.

If you decide to leave the study, you can ask that the information that was collected about you not be used for the study. Let the study staff know if you choose this. Otherwise, information that was recorded before you withdrew will still be used by the researchers for the purposes of the study, but no information will be collected after you withdraw from the study.

CAN PARTICIPATION IN THIS STUDY END EARLY?
The researcher may stop your participation in the study early, and without your consent, for reasons such as:
- You are unable to tolerate the study intervention.
- You are unable to complete all required study procedures.
- We believe it would be unsafe for you to continue to participate in the study.

If this happens, it may mean that you would not receive the study intervention for the full period described in this consent form.

If you are removed from this study, the study staff will discuss the reasons with you.

WHAT ARE THE RISKS OR HARMS OF PARTICIPATING IN THIS STUDY?
You may experience side effects from participating in this study. Some side effects are known and are listed below, but there may be other side effects that are not expected. You should discuss these with the study staff.

Less likely (5-20% of people):
In 100 people doing reactive balance training, between 5-20 may feel:

- Muscle soreness. People who start a new exercise program will often experience muscle soreness. This soreness is normal and expected with exercise, and should go away in 2-3 days. If your muscles feel sore before or during the training session, tell the study staff; they will modify the program.
- Fatigue. You might find the training or balance tests to be tiring. You will be provided regular rest breaks and can request additional breaks. You can stop the training or testing at any time if you are too tired to continue or are uncomfortable.
- Joint pain. Some people report joint pain while doing the balance training. Joint pain may be more likely for people who have a history of arthritis or joint injury. Tell the study staff if you experience any joint pain; they will modify or remove any exercises that might be causing you pain.

Rare (less than 1% of people)

- Falling. The balance training and tests are challenging to balance; there is a risk that you might not be able to recover your balance and fall. You will wear a safety harness to prevent you from falling to the floor. Additionally, the researchers can help you to regain your balance. There is a very small chance you will have an injury (such as a sprain or a bruise), even if you are caught by the safety harness.

WHAT ARE THE BENEFITS OF PARTICIPATING IN THIS STUDY?
If you agree to take part in this study, the experimental intervention may or may not be of direct benefit to you. We hope the information learned from this study will help other people with stroke in the future.

HOW WILL PARTICIPANT INFORMATION BE KEPT CONFIDENTIAL?
If you decide to participate in this study, the study staff will only collect the information they need for this study.

Your data will be shared as described in this consent form and/or as required by law and/or applicable research regulations. Records identifying you will be kept confidential and, to the extent permitted by applicable laws, will not be disclosed or made publicly available.

Authorized representatives of the following organizations may come to the hospital or be given remote access to an electronic portal (via Internet) to look at your original (identifiable) medical/clinical study records, to check that the

information collected for the study is correct and follows proper laws and guidelines. When using the electronic portal, we will share your medical record number using a secure method, so that your records are included as part of their review.

- Representatives of the University Health Network including the UHN Research Ethics Board, who oversees the ethical conduct of this study at UHN.

These individuals have completed privacy training and signed confidentiality agreements and/or are required by law to keep your information confidential.

Whether on-site or remotely, UHN makes all efforts to ensure that your information is shared in a way that is secure and private (encrypted). However, any electronic communication carries some risk of third parties gaining unauthorized access to information.

Studies involving humans sometimes collect information on race and ethnicity as well as other characteristics of individuals because these characteristics may influence how people respond to different interventions. Providing information on your race or ethnic origin is voluntary.

If the results of this study are published, your identity will remain confidential. It is expected that the information collected during this study will be published in scientific journals and presented to the scientific community at meetings.

Even though the likelihood that someone may identify you from the study data is very small, it can never be completely eliminated.

The researcher will keep any personal health information about you in a secure and confidential location for 10 years.

Your participation in this study will also be recorded in your medical record at this hospital.

Research information in shared clinical records
If you participate in this study, information about you from this research project may be stored in your hospital file and in the UHN computer system. The UHN shares the patient information stored on its computers with other hospitals and health care providers in Ontario so they can access the information if it is needed for your clinical care. The study team can tell you what information about you will be stored electronically and may be shared outside of the UHN. If you have any concerns about this, or have any questions, please contact the UHN Privacy Office at 416-340-4800, x6937 (or by email at privacy@uhn.ca).

The video recordings will be stored in a secure location and viewed only by members of the research team.

WILL FAMILY DOCTORS/HEALTH CARE PROVIDERS KNOW WHO IS PARTICIPATING IN THIS STUDY?
Your family doctor/health care provider will not be informed, specifically by the study team, that you are taking part in the study. However, if your family doctor/health care provider is part of UHN, then they will be able to see that you are taking part of the study, via our medical record system (Epic). You can choose to let your family doctor/health care provider know on your own, if you like.

WILL INFORMATION ABOUT THIS STUDY BE AVAILABLE?
A description of this clinical trial will be available on http://www.clinicaltrials.gov, as required by U.S. Law. This Web site will not include information that can identify you. At most, the Web site will include a summary of the results. You can search this Web site at any time.

Study results will be shared with you if/when they become available after the entire study is completed. It is expected that this may take a number of years. The results will be shared with you based on your preferred communication method indicated in UHN's medical record system. Please talk to the study staff if you have any questions about the results.

WHAT IS THE COST TO PARTICIPANTS?
Taking part in this study may result in added costs to you. For example:
- There may be costs associated with hospital visits. For example, parking or transportation, or snacks/meals during your stay.

ARE STUDY PARTICIPANTS PAID TO THE IN THIS STUDY?
If you decide to participate in this study, you will receive $100 total for completing the study. You will receive $80 after completing the balance training and testing, and another $20 after completing the 1 year of falls reporting.

You will be reimbursed for TTC fare (maximum: $6.70 per visit) or parking costs (maximum: $20 per visit) that result from attending the study visits.

WHAT WILL HAPPEN IF PARTICIPANT IS INJURED DURING THE STUDY?
If you are harmed as a direct result of taking part in this study, you will be referred for appropriate medical care.

WHAT ARE THE RIGHTS OF PARTICIPANTS IN A RESEARCH STUDY?
You will be told, in a timely manner, about new information that may be relevant to your willingness to stay in this study.

Your rights to privacy are legally protected by federal and provincial laws that require safeguards to ensure that your privacy is respected.

By signing this form you do not give up any of your legal rights against the study doctor, sponsor or involved institutions for compensation, nor does this form relieve the study doctor, sponsor or their agents of their legal and professional responsibilities.

WHOM DO PARTICIPANTS CONTACT FOR QUESTIONS?

If you have questions about taking part in this study, or if you experience a research-related injury, you can talk to the study staff. These people are:

David Jagroop, MHSc, CSEP-CEP
Clinical Research Analyst
Phone: 416-597-3422 ext 7614

Cynthia Danells, MSc, BScPT
Clinical Research Coordinator
Phone: 416-597-3422 ext 7884

Avril Mansfield, R.Kin, PhD
Senior Scientist
Phone: 416-597-3422 ext 7831

If you have questions about your rights as a participant or about ethical issues related to this study, call the Chair of the University Health Network Research Ethics Board (UHN REB) or the Research Ethics office number at 416-581-7849. The REB is a group of people who oversee the ethical conduct of research studies. The UHN REB is not involved in the study at all. Everything that you discuss will be kept confidential.

You will be given a copy of this signed and dated consent form prior to participating in this study.

TITLE: **Optimal intensity of reactive balance training post-stroke: a randomized controlled trial**

CONSENT
All of my questions have been answered
- I allow access to medical records as explained in this consent form
- I do not give up any legal rights by signing this consent form,
- I agree to take part in this study.

______________________	____________________	_______________
Signature of participant	PRINTED NAME	Date

______________________	____________________	_______________
Signature of person conducting the consent discussion	PRINTED NAME & ROLE	Date

The following attestation must be provided if the participant is unable to read:

If the participant is assisted during the consent process, please check the relevant box and complete the signature space below:

☐ The consent form was read to the participant. The person signing below attests that the study as set out in this form was accurately explained to the participant, and any questions have been answered.

__________________________	______________	____________________
PRINT NAME of witness	Signature	Date

____
Relationship to participant
