# Supplementary material for: Investigating the optimal reactive balance training intensity in people with chronic stroke: Study protocol for a randomized control trial
Source: PLoS One. 2025 Sep 9;20(9):e0327937. doi: 10.1371/journal.pone.0327937 (PMC12419664; doi:10.1371/journal.pone.0327937)
Supplement: S4 File — (PDF) [file pone.0327937.s004.pdf]

WHO Trial Registration Dataset

1. Trial registration: ClinicalTrials.gov ID, NCT06555016
2. Date of registration: 12 August 2024
3. Secondary identification numbers: Not applicable.
4. Sources of monetary or material support: This study is supported by the Heart and Stroke foundation of Canada (G-24-0038169).
   NM hold a doctoral personnel award from the Heart and Stroke Foundation of Cananda and Brain Canada Foundation.
5. Primary sponsor: Avril Mansfield
6. Secondary sponsor: Nigel Majoni, Elizabeth Inness
7. Contact for public queries: Avril Mansfield; address: 550 University Ave, Toronto, ON, M5G 2A2; tel: 416-597-3422 ext 7831; e-mail: avril.mansfield@uhn.ca
8. Contact for scientific queries: Avril Mansfield; address: 550 University Ave, Toronto, ON, M5G 2A2; tel: 416-597-3422 ext 7831; e-mail: avril.mansfield@uhn.ca
9. Public title: Optimal Intensity of Reactive Balance Training Post-stroke
10. Scientific title: Investigating the optimal reactive balance training in people with chronic stroke: study protocol for a randomized controlled trial
11. Countries of recruitment: Canada.
12. Interventions: Prior to training, we will determine each participant's multi-step-threshold in the forward, left and right directions. The multi-step threshold is the largest perturbation magnitude for which participants can recover their balance with only one step. Once completed, participants will be then put into one of the three intervention groups. The high intensity reactive balance training, the moderate intensity reactive balance training, and the walking control groups. The two reactive balance training groups with perform 36 multi-directional perturbations, while the walking group with perform 36 walking trials. The high intensity reactive balance training group will perform their training perturbations at 150% of their multi-step threshold (e.g., if a participant has a multi-step threshold of 3.00m/s^2, then they would perform their perturbations at 4.50m/s^2). The moderate intensity group will perform their perturbations at their multi-step threshold. At the end of every session, participants will also perform retention trials where they will be exposed to new perturbations where they have not been trained (e.g., backward perturbations). Each training session will be 1 hour in length, for four consecutive days. The number of perturbations completed in a session may be reduced, or sessions may be skipped/terminated, if participants' report adverse outcomes (e.g., pain or fatigue). If participants are fearful of the training interventions the physiotherapist will decide to reduce the intensity regardless of the stepping responses to ensure the training is tolerable. All modifications to training will be made by the physiotherapist. Participants will come back one week later for a post assessment for only the novel perturbations.
13. Key inclusion and exclusion criteria: Inclusion criteria: Adults who have chronic stroke (> 6 months post stroke). Exclusion criteria: have cognitive impairment or severe language communication (e.g., MoCA), unbale to stand independently without upper-limb support for more than 30 seconds or walk independent for more than 10 metres, have any other neurological condition that may affect balance control, have as osteoporosis, activity restrictions due to cardiac event/surgery, or severe spasticity in the lower extremity, and currently attending in- or out-patient physiotherapy or supervised exercise.
14. Study type: An assessor-blinded randomized controlled trial.

15. Date of first enrolment: 11 November 2025.
16. Target sample size: 63.
17. Recruitment status: Currently recruited and 5/63 people.
18. Primary outcomes: To assess transfer of learning using number of steps taken to recover their balance following the novel perturbation.
19. Secondary outcomes: Adverse events such as injures, pain, fear, and anxiety related to the training interventions. Mechanisms of improved reactive stepping, functional balance and falls efficacy.
